# Study design to evaluate a web-intervention to prevent alcohol and cannabis-impaired driving and use among adolescents in driver education

**DOI:** 10.1186/s13722-023-00373-2

**Published:** 2023-03-24

**Authors:** Karen Chan Osilla, Elizabeth J. D’Amico, Rosanna Smart, Anthony Rodriguez, Katherine Nameth, Justin Hummer

**Affiliations:** 1 Department of Psychiatry and Behavioral Sciences, Stanford University School of Medicine, 401 Quarry Road, Palo Alto, CA 94305 USA; 2 RAND Corporation, 1776 Main Street, PO Box 2136, Santa Monica, CA 90407‑2138 USA; 3 RAND Corporation, 20 Park Plaza # 920, Boston, MA 02116 USA

**Keywords:** Adolescents, Web intervention, Impaired driving, Alcohol, Cannabis, Marijuana, Prevention

## Abstract

**Background:**

Alcohol and cannabis are the most commonly used substances among adolescents in the U.S. The consequences related to using both substances together are significantly higher relative to use of either substance alone. Teens’ propensity to engage in risky driving behaviors (e.g., speeding, rapid lane changes, and texting) and their relative inexperience with the timing and duration of cannabis’ effects puts them at heightened risk for experiencing harms related to driving under the influence. Use of alcohol and cannabis peak at age 16, the legal age teens may apply for a provisional driver’s license in some states. Targeting novice teen drivers prior to licensure is thus an ideal time for prevention efforts focused on reducing alcohol and/or cannabis initiation, use, and impaired driving.

**Methods:**

The current study proposes to evaluate the efficacy of webCHAT among 15.5 to 17-year-old adolescents (n = 150) recruited at driver education programs. WebCHAT is a single session online intervention that aims to prevent alcohol and cannabis use and risky driving behaviors. We will recruit adolescents enrolled in driver education programs, and stratify based on whether they used cannabis and/or alcohol in the past 3 months (60% screening negative and 40% screening positive). All participants will receive usual driver education and half will also receive webCHAT. We will test whether webCHAT in addition to usual driver education reduces alcohol and/or cannabis initiation or use and reduces risky driving attitudes and behaviors (intent to drive after drinking/using, riding as a passenger with someone who drank/used) compared to teens in usual driver education over a 6-month period. We will also explore whether variables such as beliefs and perceived norms serve as explanatory mechanisms for our outcomes.

**Discussion:**

The study has the potential to promote public welfare by decreasing adolescent initiation and use of cannabis and alcohol and reducing risky driving behaviors that can have substantial monetary, personal, and social costs. The study recruits adolescents who are at risk for substance use as well as those who are not and it is delivered remotely during a teachable moment when adolescents receive driver education.

*Trial registration* This study was registered with ClinicalTrials.gov on July 13, 2021 (NCT04959461). https://clinicaltrials.gov/ct2/show/NCT04959461

As the policy landscape for cannabis has changed, adolescents increasingly view cannabis as a substance that carries little or no risk of harm [1–5]. As of 2022, 37 U.S. states allow medical cannabis use, and 21 states have passed initiatives to legalize the use and sale of cannabis for nonmedical or recreational purposes [6]. Legalization of nonmedical cannabis sales and marketing requires new approaches for preventing adolescent initiation and impaired driving. As public opinion shifts in favor of cannabis legalization [7, 8], teens are increasingly exposed to messages purporting that cannabis is not a drug or is less harmful than other substances [3, 4]; and exposure to cannabis advertising is strongly related to increased cannabis use, positive beliefs about the drug, intent to use in the future, and negative consequences [9]. The percentage of adolescents in the U.S. who perceive no risk from using cannabis once or twice a week has increased sharply in recent years [10, 11]. Among U.S. high school students, perceived safety of cannabis use is at its highest rate in two decades, with almost 60% of 10th graders reporting beliefs that smoking cannabis regularly (> 1–2 times/month) does not carry great risk [10, 11].

Alongside these shifting perceptions, an increasing proportion of adolescents who drink alcohol are also reporting cannabis use [12]. Nationwide, cannabis use prevalence among adolescents has remained relatively stable despite the sharp decline in risk perceptions among this population [13–15]. Underage drinking and impaired driving fatalities have also seen modest decreases in recent years [16–18]. However, the public health benefits from these declines may be partially offset by increases in the number of adolescents who use both substances [12]. Alcohol and cannabis remain the most commonly used substances among high-school aged adolescents in the U.S. [10, 19], and they are commonly consumed together. About 20% of 12–17 year-olds who reported drinking alcohol in the past month also reported simultaneously using cannabis within 2 h [11]. Adolescent use of both substances, particularly simultaneous use, is associated with serious consequences compared to using either substance alone, including greater risk of substance use disorder [20, 21], binge drinking [22], and unsafe driving [23].

Of critical concern when alcohol and cannabis are used simultaneously is their combined effect on driving [24–26], and adolescent drivers may be at disproportionate risk compared to more experienced drivers [23, 27]. When used simultaneously (use within the same occasion so the effects overlap), the risk of driving impairment (e.g., reaction time) significantly increases compared to either substance alone [24, 25, 28–31]. The combined effects on psychomotor and cognitive functions have additive, or possibly synergistic, effects on impairment [28, 31–34] Of note, the additive effect of combining alcohol and cannabis is most pronounced at low levels of blood alcohol concentration [32]. Adolescents may be particularly susceptible to these risks because of their high rates of simultaneous use and the unique social contexts in which polysubstance use often occurs (e.g., in a car or driving) [27], further raising the likelihood of vehicular accidents [23]. A recent study with adolescents found that each occasion of simultaneous use was associated with a more than threefold increase in driving under the influence (DUI) or riding with an impaired driver [35].

While simultaneous use seems to confer the greatest risk on driving, concurrent use of alcohol and cannabis (use of both substances in the past month, but on different occasions) also places teens at risk for greater consequences. Among high school seniors across the U.S., rates of unsafe driving behaviors (receiving tickets; accidents while driving) peak when alcohol and cannabis are used simultaneously, followed by concurrent use, and then drinking alcohol alone [23]. 

Adolescents’ propensity to engage in risky driving behaviors (e.g., speeding, rapid lane changes, and texting) [36–38] combined with their relative inexperience with the timing and duration of cannabis’s effects [39, 40] puts them at heightened risk for experiencing harms related to impaired driving. Interventions aimed at preventing alcohol-impaired driving among adolescents have contributed to reducing prevalence rates of underage drinking and driving [16–18] but these may not be effective for addressing cannabis-impaired driving as cannabis expectancies may function differently than those for alcohol [41] and may require more targeted approaches [42–44]. Further, driving under the influence of alcohol alone, cannabis alone, or both substances together involve different patterns of use, motivations, social contexts, and cultural reasons [45–48]. The state of knowledge about how cannabis affects driving safety also pales in comparison to that of alcohol [49], which has for decades been widely recognized to impair driving performance and increase crash risk even at low levels [46, 50–52]. Thus, whereas most adolescents clearly understand the risks associated with drinking and driving [53], they are often exposed to contradictory information regarding potential harms of cannabis on driving safety [54].

Existing web-based intervention (WBI) studies that address alcohol and cannabis use tend to focus on older adolescents or adults who already use, target a single substance, and do not exclusively focus on impaired driving. Providing an early intervention to teens before more serious consequences develop is essential; by the time an individual receives a first-time DUI, a brief intervention may not be as efficacious as they may have already developed a substance use disorder [55]. Thus, it is imperative to use WBIs to target teens *before* they receive a DUI and experience life-changing consequences. Alcohol and cannabis WBIs have been widely tested and demonstrated efficacy across a variety of settings, including at home [56–63], in schools [64–69], student health centers [70], and in primary care settings [71, 72]. However, the existing WBI literature is limited in three main ways. First, existing WBIs have mostly targeted at-risk college students and adults who already drink or use cannabis, and these interventions may not be developmentally appropriate for a younger population who has not initiated or may not yet be experiencing substance-related consequences [73–75]. Second, with few exceptions [56], existing WBIs primarily focus on alcohol *or* cannabis, and do not focus on both substances during the developmental period in which impaired driving is most risky. A final limitation is that no WBIs have been disseminated in driver education settings, despite online training becoming the predominant mode of education for newly licensed drivers, and approved in 16 states [76].

Driver education is an important time to intervene and can be a teachable moment as teens are focused on learning driver-related messaging (e.g., how to drive safely) because they are motivated to obtain their license and prevent accidents. Opportune or teachable moments can be interactions during specific events or context where the focus on learning is enhanced and may cause behavior change [77].

Our study advances the field substantially by developing and evaluating a WBI to prevent alcohol and cannabis use using a developmentally appropriate, opportunistic time when teens are learning to drive. We adapted an existing in-person effective intervention for teens called CHAT to create webCHAT. CHAT was developed for use in primary care for at risk teens, and is a 15-min in-person intervention that addresses both alcohol and cannabis use [84]. At 3- and 6-month follow up, teens that received CHAT reported lower perceived peer use of alcohol and cannabis compared to usual care; in addition, at 6 months, they reported fewer negative consequences from alcohol (e.g., got into a fight, felt really sick) compared to usual care. At 12 months, CHAT teens continued to report lower perceived use of both substances, and also reported fewer alcohol and cannabis-related consequences (e.g. missing school, getting into trouble) compared to usual care [95]. Furthermore, those at higher risk (e.g., with alcohol or cannabis use disorder) who received CHAT reduced their alcohol and cannabis use and experienced fewer consequences 1 year later [95]. In sum, we found that this 15-min intervention had long-term positive effects for lower and higher risk teens on both alcohol and cannabis use and consequences.

Our webCHAT intervention will address alcohol and cannabis use alone, and simultaneous and concurrent use of these substances, as many adolescents are likely to use both substances. Moreover, the concept for this WBI is that it will apply to all teens, regardless of their prior experience using either substance. Some teens may not have initiated cannabis or alcohol use, or if they are using, would likely not yet have experienced substance-related consequences or driven under the influence. WebCHAT can therefore be incorporated into driver education for all teens regardless of their use patterns.

## Specific aims and hypotheses

The specific aims of this project are to: (1) assess the efficacy of adding webCHAT to usual driver education compared to usual driver education (i.e., webCHAT vs. no webCHAT) on alcohol and cannabis initiation/use and driving attitudes and behaviors at 3 and 6 months after baseline; (2) explore whether variables such as self-efficacy and perceived norms serve as explanatory mechanisms for our outcomes. We hypothesize that teens who receive webCHAT will report reduced alcohol and/or cannabis initiation or use, reduced risky driving attitudes (intent to drive after drinking/using) and risky passenger behaviors (e.g., passenger with someone who drank/used) compared to teens in usual driver education.

## Methods/design

### Overview of study procedures

All procedures have been approved by Stanford’s Institutional Review Board (IRB) and will be renewed annually. Protocol modifications will be reviewed by key personnel and reported to the funder and our IRB. The current paper describes a parallel group two-arm randomized controlled trial pilot test of webCHAT versus no webCHAT among teens attending driver education. Teens will be recruited from at least two driver education schools. If teens are eligible and have a parent/guardian who consents to their teen’s participation, teens will be randomized to receive webCHAT or not and followed for 6 months.

### Study setting

This study will take place in at least two driver education programs in Michigan and Colorado. Both states have graduated driver licensing laws where teens succeed in stages regarding their driving privileges (e.g., nighttime driving and passenger restrictions) [78]. If under age 16, adolescents need to receive a driver’s permit, complete 50 h of supervised driving, and complete driver education classes prior to applying for their license [90]. Driver education options vary and typically consist of either in-person or online instruction as well as behind-the-wheel training in the car with an instructor. In both Michigan and Colorado, the behind-the-wheel training is a minimum of 6 hours. Parents/guardians are offered a parent orientation before adolescents start behind-the-wheel training.

Both states have legalized medicinal cannabis use for those 18 and older and recreational use for those 21 and older; however, cannabis-impaired driving remains a criminal offense. If someone is driving under the influence of alcohol/cannabis with a driver’s permit, their permit is revoked for 12 months and needs to be reinstated for a subsequent 12 months prior to applying for their license. If driving under the influence with a valid license, the driver is charged with a misdemeanor depending on the specific cannabis laws in each state.

### Participants

Eligible participants will be 180 adolescents aged 15.5 through 17 (inclusive) who are within a month completion of their 6-h behind-the-wheel training at one of the participating driver education schools and have a parent/guardian that consents their participation in the study. We focus on adolescents in this age range because students often start their driver’s training prior to age 16, must be 16 or older to apply for their driver’s license, and cannabis and drinking peaks at 16 [79, 80]. We also want to recruit adolescents just prior to them being eligible to apply for their driver’s license at age 16 to capitalize on a teachable moment when they are still in driver education and may be more receptive to learn about safe driving. In addition, this timing allows us to recruit teens who will soon take their licensing exam so that we may measure driving behaviors at follow-up for those that pass their licensing exam. Finally, we extend the age range to those who are 17 acknowledging that some adolescents may be waiting until they are older to apply for their license [81], yet they are still at-risk for the consequences associated with novice driving and substance use.

### Procedures

**Fig. 1:**
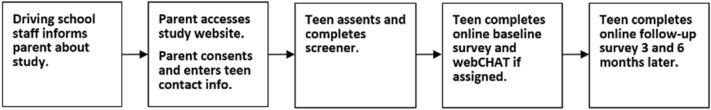
Study flow

Parents/guardians will be asked to consent for their teen to participate at parent orientation meetings at the driving schools or through promotional materials that driving school staff send. Interested parents/guardians will then be asked to reach out to the study coordinator and share when their teen is expected complete their behind-the-wheel training. If within a month of completion, the parent will be invited to review consent materials, consent online, and provide teen contact information for their teen to participate. If beyond a month, the parent will be asked if they would like to place their teen’s name on a study waitlist to be contacted when their teen is close to completion. For parents that consent for their teen to participate, a research staff member will send the teen a text and/or email that includes a unique URL, and username and password for the teen to learn about the study and assent for their participation. If the teen assents, research staff will verify when they will complete their behind-the-wheel training. Then, the teen will be invited to complete an online screener to determine risk, a baseline survey, randomized to condition, webCHAT session and post-session survey if randomized, and an online follow-up survey 3- and 6-months later (see Fig. 1). We will stratify our sample by risk with 60% screening negative and 40% screening positive based on whether they have used alcohol (more than a few sips) and/or cannabis in the past 3 months as current use is associated with greater problems [12]. This stratification is based on our power estimates to determine intervention effects within those who use and those who have not initiated (see “Power” below).

Teens will be encouraged to keep their profile and password to themselves. Upon completing the baseline survey, teens will not be able to see their prior responses to their baseline survey so that any unintended breaches of confidentiality from re-accessing survey information is prevented. Teens will also be encouraged to view the webCHAT intervention in a private setting using earphones. Teens can complete webCHAT and their online surveys from any device including their smartphone from anywhere where they have internet access. Teens will be asked to complete the session within 1 week of completing the baseline survey. A series of reminder emails, texts, and/or phone calls will be sent to those who do not complete the session within the first week. The terminal duration for nonresponse will be 4 weeks, at which time the participant will be informed that they will no longer be permitted to complete the remainder of the study. After the session, there will be a short satisfaction survey assessing acceptability and usability of the session (e.g., on a 1–10 scale, how helpful/unhelpful was the session?). All participants will receive $25 for the baseline survey, $40 for the 3-month survey, $40 for the 6-month survey, with a $25 bonus if all three surveys are completed. WebCHAT participants will also receive an additional $5 for completing their satisfaction survey following the intervention. Teens who are assigned to usual driver education will be offered an opportunity to complete webCHAT after their 6-month follow-up survey.

**Fig. 2:**
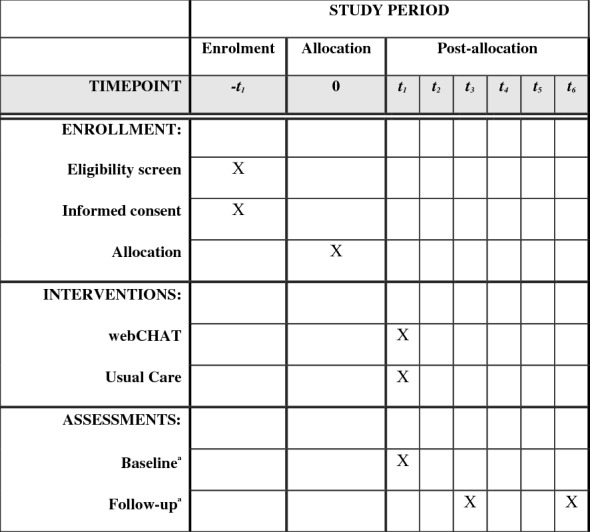
SPIRIT flow diagram. ^a^Primary outcomes (alcohol/cannabis initiation/use, driving attitudes), secondary outcomes (driving behaviors, substance-related consequences), and mediators (beliefs and perceived descriptive norms)

A total of 188 teens will be recruited for the study, resulting in about 150 teens at 6-month follow-up (assuming 80% retention). To ensure robust follow-up rates, we will obtain detailed information at baseline on how to reach participants and use proven methods to minimize attrition, including an in-person baseline interview to build rapport, obtaining multiple contacts (friends/families/service providers) at baseline for individuals who would know a participant’s whereabouts, and phone/mail/text/social media reminders prior to follow-up [95] (see Fig. 2).

### Randomization

Upon completion of the baseline survey, teens will be randomly assigned (computer-generated) to webCHAT vs. no webCHAT using permuted block randomization with random size blocks. This ensures the number of people allocated to each group is approximately equal throughout recruitment [82]. Participants within each group will be stratified (40% alcohol and/or cannabis use in the past 3 months, 60% no use). Participants will be informed of randomization immediately after completing their baseline survey. Surveys and webCHAT are completed entirely online without researcher involvement, and thus, research staff will be blinded to intervention assessment.

### Usual driver education

Driver education typically has two main phases: (1) Classes that need to be taken before they can apply for their driver’s permit and (2) behind-the-wheel training with an instructor before they can take their licensing exam. Classes are online or in-person and consist of between 24 and 34 h of curriculum on how to drive to receive their driver’s permit [83]. Behind-the-wheel training typically consists of six 1-h sessions of supervised driving with an instructor that are spaced apart by time (a couple weeks to a month) or driving practice time (20 h) to allow for supervised driving practice with a parent/guardian between sessions. Classes can consist of training videos, interactive exercises, and quizzes. In one of the participating driver education schools, one of the online classes is devoted to impaired driving and discusses (1) alcohol and its effects (blood alcohol content, myths/facts about alcohol, driving simulation video showing the effects of drinking and driving, statistics regarding alcohol crashes, how alcohol affects judgement and muscle control, refusal skills); (2) effects of cannabis (effects on judgement and reaction time, information on driving simulation studies that show that cannabis affects driver’s (in)ability to recognize and respond to dangerous situations, how cannabis has greatest impact on new drivers, information on the synergistic effect of combining drugs and alcohol on collision); (3) effects of other drugs (stimulants, depressants, hallucinogens) on driving; (4) distracted and drowsy driving; and (5) how emotions can impair driving (e.g., road rage and aggressive driving). The online program is interactive including audio narration, video examples, quizzes, and interactive exercises; however, these videos are quite different from webCHAT. Specifically, the training is not personalized to the teen’s experience with using alcohol or cannabis, it does not utilize MI language, does not discuss the pros and cons of use and impaired driving, and does not address cannabis expectancies or effects of using alcohol and cannabis together.

### webCHAT intervention

Our intervention content draws from three established theories to prevent risk behaviors [84]: (1) expectancy theory to address positive and negative expectancies related to alcohol and cannabis use and driving behavior [85]; (2) social learning theory to address peer modeling and normative beliefs [43, 86, 87]; and (3) decision making theory to address decisions about engaging in impaired driving and resistance self-efficacy [88].

**Fig. 3:**
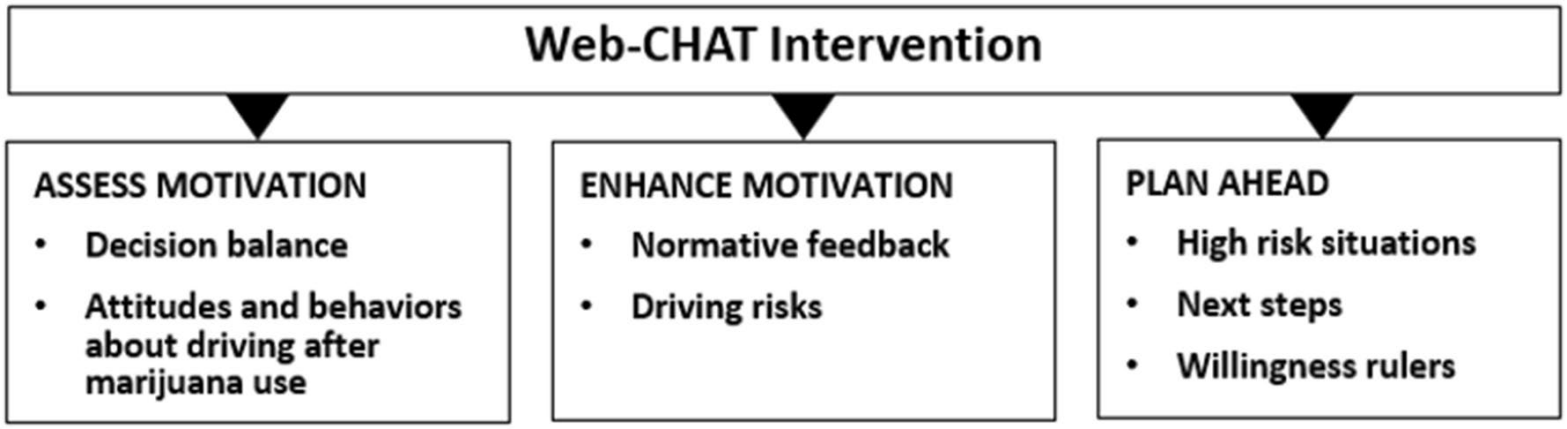
webCHAT intervention

The intervention uses a motivational interviewing (MI) style and process [89] Consistent with the four fundamental processes of MI, *engagement* will be promoted through an intervention “tone” that emphasizes autonomy, collaboration and evocation (e.g., providing choices, statements like, “it’s up to you”). *Focusing* will include teen vignettes that help teens learn about high-risk impaired driving situations. *Evoking* strategies will include personalized feedback, targeted open questions and selective reinforcement of responses (e.g., willingness and confidence rulers). Finally, *planning* will be addressed through activities where teens identify coping strategies they would use in high-risk situations (See Fig. 3).

The intervention was adapted to smartphone viewing and focuses on three phases (see Fig. 1): assessing motivation to change, enhancing motivation, and planning ahead. As with our other WBI work [55, 90, 91], an on-screen narrator uses MI-based language [89] to help engage the participant by building self-efficacy and enhancing intrinsic motivation to change.

**Fig. 4:**
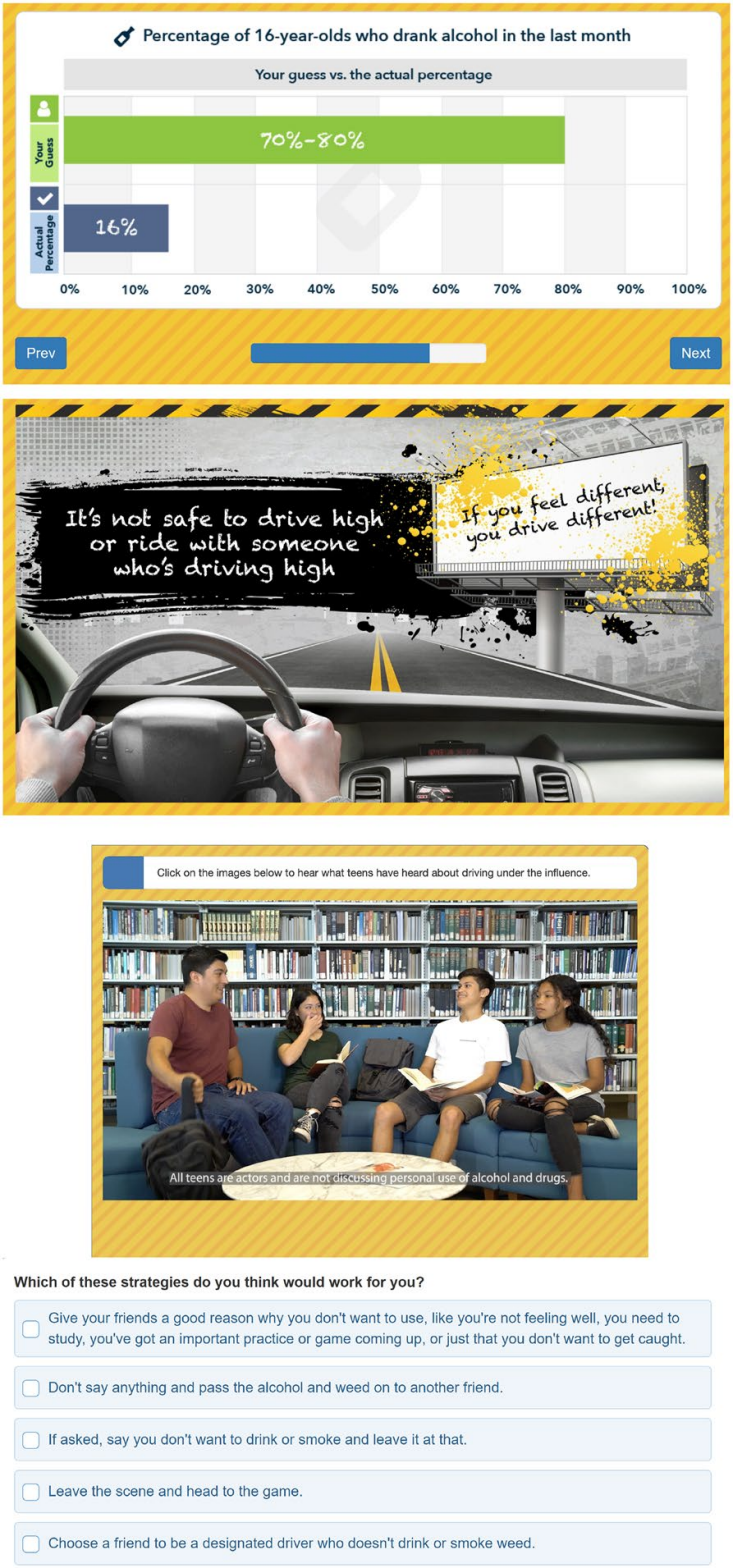
Screenshots of webCHAT

In the first phase of the intervention, teens will view a brief video to orient them to the intervention, explain what they will see next (decisional balance, norms), and suggest ways they could use what they learn to prevent future cannabis, alcohol use, and impaired driving. After this initial video, subsequent audios, videos, and interactive exercises will review intervention topics and are tailored based on teens’ reported alcohol and cannabis use. By utilizing an interactive WBI, we hope to enhance their experience and involvement with the content and support their behavioral changes (Fig. 4) [92].

Similar to our previous WBIs for young adults [55, 90, 93, 94], the intervention uses the teen’s baseline responses to highlight the good and not-so-good things they have experienced or heard about cannabis and alcohol. Video/audio testimonials from teen actors will be shown at key points to enhance the intervention’s credibility [94] and to illustrate decision making in common high risk situations (e.g., what teens can do if they need a ride home from a party where their friends are using alcohol, cannabis, or both). Because participants will have completed their baseline survey prior to the intervention, we will use tailored messaging (e.g., programming logic and if/then statements to personalize intervention content to participant’s responses) to deliver audios, videos, and text corresponding to whether they are abstinent or use alcohol and/or cannabis. Intervention content will thus depend, in part, upon what substances the teen reports using at baseline [84, 95, 96]. Teens that report only alcohol or only cannabis use will get personalized feedback and decisional balance information on that specific substance. They will also get information on simultaneous use of cannabis and alcohol and their effects on driving. For teens who report both alcohol and cannabis, the narrator will highlight the teen’s baseline responses regarding what they like and dislike about using both and ask them to explore the positive and negative beliefs they have about both and their effects on driving. For teens that report no use, the intervention content will focus more generally on making healthy choices around use of substances and driving, taking a more preventive approach summarizing what they reported at baseline regarding what they have heard about the good and not-so-good things about alcohol and/or cannabis use. We will also explore their positive and negative beliefs about cannabis and driving after drinking and cannabis use (e.g., What have you heard about the effects of cannabis and alcohol? How about how it affects driving?).

The second phase of the intervention will focus on enhancing motivation. Using the teen’s baseline survey responses, we will display personalized normative feedback, which has been shown to be effective for both adolescents that use and those that abstain [97, 98]. We will also use teen vignettes where they discuss the risks of driving after alcohol and cannabis (alone and using both alcohol and cannabis together). The narrator will summarize teaching points on how cannabis affects the brain and their ability to multi-task, the effects of using both alcohol and cannabis on driving, and how riding with a driver who has used substances is also risky.

The final phase of the intervention will focus on protective behavioral strategies by using teen vignettes to discuss three common high-risk situations associated with impaired driving (e.g., football game, party, friend’s house). Teen actors will role-play common reactions that teens have in each of these situations to validate different perspectives. Each situation will include a teen promoting protective behavioral strategies and the narrator reinforcing these teaching points. After each situation, participants will select which strategies they think they would try before watching the discussion on the next situation. Finally, we will summarize the strategies participants chose and ask teens to choose a number between 0 (not willing) and 10 (very willing) indicating their willingness and confidence to try a strategy to prevent impaired driving. Teens will be asked to type a number and asked why they did not choose a lower number in order to facilitate change talk. At the conclusion of the intervention, the narrator will summarize the discussion.

### Measures

Our primary outcomes will include alcohol and/or cannabis initiation or use, intent to drive after drinking/using, and frequency of being a passenger with someone who drank/used. We will assess frequency of alcohol, cannabis, and alcohol/cannabis co-use (concurrent and simultaneous) in the past 3 months [12], likelihood of future driving after alcohol and/or cannabis use from the Behaviors and Attitudes Drinking and Driving Scale [99] that asks participants how likely they are to drive short (a few blocks to a mile), medium, and long (over 20 miles) distances after drinking/cannabis use, and how often they were a passenger in a car or other vehicle with a driver who had been drinking alcohol and/or using cannabis [100, 101].

Our secondary outcomes will include driving behaviors and alcohol and cannabis consequences (if they report use). Because this is a young sample learning how to drive, rates of impaired driving may be low, but we will still measure how often they drove a car, motorcycle or other vehicle after using cannabis, alcohol, and/or both [102]. We will ask if they received any traffic violations (e.g., speeding tickets, alcohol/drug violations) or were in any car accidents, and we will obtain a measure of driving behavior (e.g., miles driven in a typical week), whether they have passed their licensing exam, and non-substance related risky driving behaviors (e.g., texting while driving, driving over the speed limit, having more than the allowed amount of passengers) as possible control variables. We will also assess alcohol and cannabis-related consequences in the past 3 months using items from the Marijuana Consequences Questionnaire [103] and other well-established measures [96, 104]. We will use ten items for alcohol [96] (e.g., missed school or work, passed out) and six for cannabis (e.g., got into trouble at school or home, had difficulty concentrating), and we will also adapt these items to assess consequences from simultaneous alcohol and cannabis use.

#### Mediators

We will assess whether beliefs and perceived descriptive norms mediate the intervention’s effect on outcomes. Nine items will assess positive and negative beliefs about alcohol and cannabis [105, 106]. We will also assess beliefs about harm using three items from the Monitoring the Future survey that asks how much harm is associated with using cannabis once or twice, occasionally, and regularly [2]. We will adapt this measure to assess beliefs about co-use. Participants will be asked perceived descriptive norms by asking them to think about a group of 100 teens their age and indicate how many students they believed had used alcohol, cannabis, and both substances at least once per month. Response options will range from 0 to 100, with multiples of 10 as anchors [107].

### Power

We draw on our in-person CHAT trial that had standardized effect sizes ranging from 0.31 to 0.86 for continuous measures of alcohol/cannabis use frequency, number of friends who use alcohol/cannabis, alcohol/cannabis intentions, and alcohol/cannabis-related consequences [95]. For a continuous outcomes, the necessary sample size depends on the correlation between the baseline and outcome measures, and assumes this multiple correlation will equal 0.6. Using a two-tailed test (α = 0.05), with a moderate standardized effect size of (Cohen’s *d*) 0.50, we estimate 80% power will be achieved with a sample size of 24, thus, we are more than adequately powered with our proposed follow-up sample of 150. Within conditions (e.g., webCHAT) and given the recruitment proportions of those who screen positive and negative, we have 80% power to detect an effect size of 0.57 between adolescents who score positive and negative within conditions (e.g., within webCHAT) on primary and secondary outcomes with a sample size of 74 in each group. Between conditions (webCHAT vs no webCHAT) we have 80% power to detect a moderate effect size of 0.50 with an overall sample of 96.

For mediation analyses, a sample size of 148 has 80% power to detect an indirect effect when δ = 0.26 (i.e., effect of the intervention on the mediator) and β = 0.26 (i.e., effect of the mediator on the outcome) when using the bias-corrected bootstrap method [108, 109]. While effects of this magnitude are not necessarily expected, these magnitudes are reasonable considering past findings. We are therefore confident in our ability to test for and identify mediated effects [110].

### Data management and analysis plan

Analyses will use the standard intent-to-treat (ITT) approach. Our ITT approach will analyze participants as belonging to the group (webCHAT or no webCHAT) they were randomized to, regardless of later non-response or loss at follow-up because excluding participants who do not attend webCHAT would bias our results in favor of webCHAT and increase the Type I error rate [111].

We will examine distributions of all outcome variables to first detect evidence of sparseness for categorical data and of non-normality for continuous variables using graphical evidence, and examination of skewness and kurtosis. For sparse categorical variables, we will collapse to produce cell sizes sufficient for analysis. For continuous outcome measures that are unlikely to satisfy the normal distribution assumption, we will consider variable transformation. Balance equivalence will be evaluated by comparing variables from the baseline survey across webCHAT and no webCHAT groups to assess balance of the randomization process. ANOVA or t-tests will be used to test for comparability of groups for continuous baseline measures, and categorical methods of analysis such as chi-square tests will be used to compare groups for discrete baseline measures (e.g., gender). Any statistically significant differences will be controlled for in subsequent analyses through addition of model covariates.

To examine group differences in primary and secondary outcomes at three and 6 months, we will use a regression-based framework. To protect against inflated Type I error rates caused by multiple testing, we will carry out a multivariate test of the simultaneous effect of the intervention on outcomes at three and 6 months. Where this multivariate test is statistically significant, we will then use individual univariate tests at each follow-up. Where variables are not normally distributed, we will consider an alternative link function; for example, for outcomes which assess the number of events that have occurred, we will employ Poisson regression with standard errors adjusted for over-dispersion if necessary. Where outcome measures are continuous, we will use ordinary least squares-based estimation. In addition, biological sex will be explored and accounted for as a covariate of interest in relevant analyses.

We will conduct mediation analyses to examine whether changes in measures such as self-efficacy, norms, and beliefs are predictors of our primary self-reported outcomes. These analyses will allow us to explore predictors of behavior change, thereby improving the development of future interventions. Mediation analysis will be carried out within a structural equation modeling framework. We will first conduct a multivariate mediation test to determine if the total mediation effect is statistically significant to control Type I error rate. If the total mediated effect is significant, we will then examine the individual mediators to determine statistical significance. The estimate of the effect of the intervention on the outcome can be divided into the direct effect and the indirect (mediated) effect. For potential mediators 1 … k, we assess: ${{\text{y}}_{\text{i}}}={{\upbeta}_{0}}+{{\upbeta}_{1}}{{int}_{\text{i}}}+{{\upbeta}_{\text{k}}}{{\text{M}}_{\text{k}}}+{{\text{e}}_{\text{i}}}$ where ${\text{y}}_{\text{i}}$ represents the outcome of interest, ${\upbeta }_{0}$ is the intercept, ${\upbeta }_{1}$ the effect of the intervention (${int}_{\text{i}}$; indexed with i to indicate the intervention status of individual i) and represents the direct effect. ${\text{M}}_{\text{k}}$ represents the set of potential mediators, and ${\upbeta }_{\text{k}}$ is the effect of the mediator on the outcome. Using a structural equation modeling framework, we will simultaneously estimate the effect of the intervention on the mediators: ${\text{M}}_{\text{ki}}={\delta }_{k0}+{\updelta }_{\text{k}}{int}_{\text{i}}+{\text{e}}_{\text{I}}$ where ${\text{M}}_{\text{ki}}$ is the mediator k for individual i. ${\updelta }_{\text{k}0}$ represents the intercept for mediator k, and ${\updelta }_{\text{k}}$ represents the effect of the intervention on mediator k. The indirect effect via each potential mediator is given by the element-wise product of the vectors ${\upbeta }_{\text{k}}$ and ${\updelta }_{\text{k}}$ and the total mediation effect is ${\upbeta }_{\text{k}}\times {\updelta }_{\text{k}}{^{\prime}}$. The standard errors of the individual indirect effects will be estimated using bias-corrected bootstrapping [112, 113].

To handle missing data, we will impute missing items from scales using within-scale mean imputation. For loss to follow up, we will use full information maximum likelihood estimation or multiple imputation to obtain estimates that are consistent and unbiased [114–117].

Findings will be disseminated at professional conferences and through scientific manuscript publication. Data sharing agreements may be possible after the main project findings are accepted for publication.

### Limitations and alternate designs considered

The proposed study is limited as we are recruiting from two driver education schools in Michigan and Colorado, and therefore may not be generalizable to other programs and geographical areas. We considered recruiting only teens who report current cannabis use, but we wanted to make this program available for all teens regardless of whether they had initiated cannabis use. For those same reasons, we wanted to tailor the intervention to the web to increase dissemination potential. This online adaptation is also necessary to keep up with the aggressive pace of cannabis legalization where language and policies are evolving. We also considered recruiting only 16-year-olds to maintain a more homogenous sample and to focus on universal prevention, but the majority of teens receiving behind-the-wheel training are 15.5 years old, and we saw this as an important opportunity to focus on teens before more serious problems may develop.

## Discussion

Our proposed study advances the field substantially by evaluating a WBI to prevent alcohol and cannabis use and impaired driving among adolescents using a developmentally appropriate, interactive WBI at an opportunistic time when teens are learning to drive. Our intervention will address alcohol and cannabis use alone, and co-use of these substances. We use a public health approach for including teens with diverse use patterns. Some teens may not have initiated cannabis or alcohol use, or if they are using, have likely not yet experienced substance-related consequences or driven under the influence. Thus, this WBI can be incorporated into driver education for all teens regardless of their use patterns. This study is intended to generalize to novice drivers attending driver education, a population ideal for intervention because of the risks of novice driving and greater potential for substance-related consequences.

## Data Availability

Not applicable.

## Abbreviations

ITT: Intent-to-treat
MI: Motivational interviewing
WBI: Web-based intervention
